# Outcomes of Paediatric Glaucoma Surgery: Insights From a Five-Year Retrospective Study in Manchester

**DOI:** 10.7759/cureus.89925

**Published:** 2025-08-12

**Authors:** Elpida Kollia, Bhamy Hariprasad Shenoy, Vinod Sharma, Rehana Sadia, Jane Ashworth, Susmito Biswas

**Affiliations:** 1 Paediatric Ophthalmology, Moorfields Eye Hospital, London, GBR; 2 Paediatric Ophthalmology, Manchester Royal Eye Hospital, Manchester, GBR; 3 Ophthalmology, National and Kapodistrian University of Athens, Athens, GRC

**Keywords:** intraocular pressure, itrack, paediatric glaucoma surgery, primary congenital glaucoma, trabeculectomy bleb, trabeculotomy

## Abstract

Purpose: The objective of this study was to assess the clinical outcomes of surgical management of primary congenital glaucoma (PCG) in a cohort of children treated at Manchester Royal Eye Hospital, Manchester, United Kingdom, over a five-year period.

Methods: This retrospective observational study analysed data from 18 children (31 eyes) diagnosed with PCG between 2019 and 2024. Clinical characteristics, surgical details, intraocular pressure (IOP), optic nerve status, and the need for further surgical intervention were recorded and analysed.

Results: The cohort consisted of 14 male and 4 female children, with a mean age at diagnosis of 14 months. Thirteen children had bilateral PCG, and five had unilateral involvement. Presenting features included excessive tearing and increased corneal diameter. The average preoperative IOP was 32 mmHg. Surgical treatment led to significant reductions in both IOP and corneal diameter. Optic disc cupping reversed in most cases. Only two eyes required additional glaucoma surgery. No significant intraoperative complications were recorded.

Conclusion: Surgical intervention in paediatric glaucoma at a tertiary centre produced favourable anatomical and functional outcomes, with a low reoperation rate. Early diagnosis and timely surgical management remain crucial in preventing vision loss in children with PCG.

## Introduction

Primary congenital glaucoma (PCG) is a rare but serious cause of childhood blindness, characterised by developmental abnormalities of the trabecular meshwork that result in elevated intraocular pressure (IOP), optic nerve damage, and globe enlargement [1-3]. The condition affects males more than females and is often bilateral [3,4]. PCG has a global prevalence estimated between one in 10,000 and one in 30,000 live births in Western populations, with a higher incidence in regions with increased consanguinity [4].

Early surgical intervention is the cornerstone of treatment, typically starting with angle procedures such as goniotomy and trabeculotomy [5]. The development of illuminated microcatheter techniques, such as 360° trabeculotomy using iTrack™ (Haag-Streit UK Ltd, Bishop's Stortford, United Kingdom), has greatly improved surgical precision and efficacy in recent years [6-9].

The objective of this study was to assess the clinical outcomes of surgical interventions for primary congenital glaucoma in children treated at Manchester Royal Eye Hospital, Manchester, United Kingdom, over a five-year period. This review of our centre’s experience offers insight into patient demographics, surgical outcomes, and follow-up trends and contributes to the ongoing evaluation of surgical strategies in managing PCG.

## Materials and methods

This was a retrospective chart review of all children diagnosed with primary congenital glaucoma (PCG) and referred to Manchester Royal Eye Hospital between 2019 and 2024. The study was approved by the Manchester University NHS Foundation Trust Research Ethics Committee (protocol number: 010202024).

Inclusion criteria were a confirmed diagnosis of PCG, surgical intervention, and a minimum postoperative follow-up period of six months. Patients with secondary glaucomas or those with syndromic or acquired forms of the disease were excluded.

Data collected included each patient’s age at diagnosis, sex, laterality of disease, presenting symptoms, pre- and postoperative IOP, corneal diameter measurements, optic nerve appearance (with particular attention to cupping), surgical details, and the need for reoperation. Functional vision data, such as visual acuity or refractive error, were not available due to the retrospective nature of the dataset.

As a tertiary referral centre, this dataset includes all eligible cases managed surgically for PCG during this period. All surgeries were performed by two senior paediatric ophthalmologists, ensuring consistency in technique. IOP was measured using age-appropriate techniques, either the iCare rebound tonometer (Icare Finland Oy, Vantaa, Finland) or the Tono-Pen (Ametek Reichert Technologies, Depew, New York, United States), depending on patient cooperation and clinical setting. Optic nerve cupping was assessed via fundoscopy and imaging, where feasible.

All patients were managed using angle-based procedures (goniotomy or trabeculotomy), with trabeculectomy performed where appropriate. If Schlemm’s canal was not patent or could not be cannulated, the procedure was converted to trabeculectomy with adjunctive mitomycin-C. Patients with refractory disease were referred to the glaucoma service for valve implantation.

Statistical analysis

Descriptive statistics were used to summarise the data. Continuous variables (e.g., IOP and age at diagnosis) were reported as mean ± standard deviation (SD), while categorical variables (e.g., sex and laterality) were expressed as counts and percentages. Statistical significance was set at p < 0.05. No inferential statistics were applied due to the limited sample size [7-9].

## Results

A total of 18 patients were included in the study, accounting for 31 eyes. Thirteen patients (72%) had bilateral disease, while five (28%) had unilateral primary congenital glaucoma. There was a male predominance, with 14 boys and four girls. The mean age at diagnosis was 14 ± 2.5 months. The most common presenting symptoms were excessive tearing (epiphora) and enlarged corneal diameters. 

Table 1 summarises the surgical techniques performed, with 360° trabeculotomy (iTrack) being the most common. Table 2 presents demographic data, and Table 3 illustrates IOP reduction by surgical type.

**Table 1:** Surgical procedures performed for primary congenital glaucoma (N=31 eyes) *Haag-Streit UK Ltd, Bishop's Stortford, United Kingdom

Surgical Technique	Number of Eyes	Percentage
Goniotomy	6	19.4%
Trabeculotomy	8	25.8%
Trabeculectomy	5	16.1%
360° Trabeculotomy (iTrack*)	9	29.0%
Combined Trabeculotomy + Trabeculectomy	3	9.7%

**Table 2:** Patient demographics Data are represented as n (%), except for age at diagnosis, which is presented as mean ± SD.

Characteristic	Value
Total Patients	18 (100%)
Total Eyes	31 (100%)
Sex - Male	14 (77.8%)
Sex - Female	4 (22.2%)
Laterality - Bilateral	13 (72.2%)
Laterality - Unilateral	5 (27.8%)
Age at Diagnosis (months), mean ± SD	14 ± 2.5
Presenting Symptoms	Epiphora, Enlarged Cornea

**Table 3:** Intraocular pressure (IOP) reduction by surgical technique *Haag-Streit UK Ltd, Bishop's Stortford, United Kingdom

Surgical Technique	IOP Reduction (%), mean ± SD	Eyes Requiring Postoperative Drops, n (%)
360° Trabeculotomy (iTrack*)	62.5 ± 8.2	2 (18.0%)
Trabeculectomy	56.6 ± 9.5	5 (31.2%)

The mean preoperative IOP was 32 mmHg. All patients underwent angle-based surgical procedures performed by the paediatric ophthalmology team. Trabeculotomy was the initial procedure of choice. If Schlemm’s canal was not patent or could not be cannulated, the procedure was converted to trabeculectomy with adjunctive mitomycin-C. Goniotomies were performed primarily between 2019 and 2020. With the advent and growing availability of the iTrack microcatheter system, 360° trabeculotomy became the preferred technique in subsequent years.

**Figure 1:**
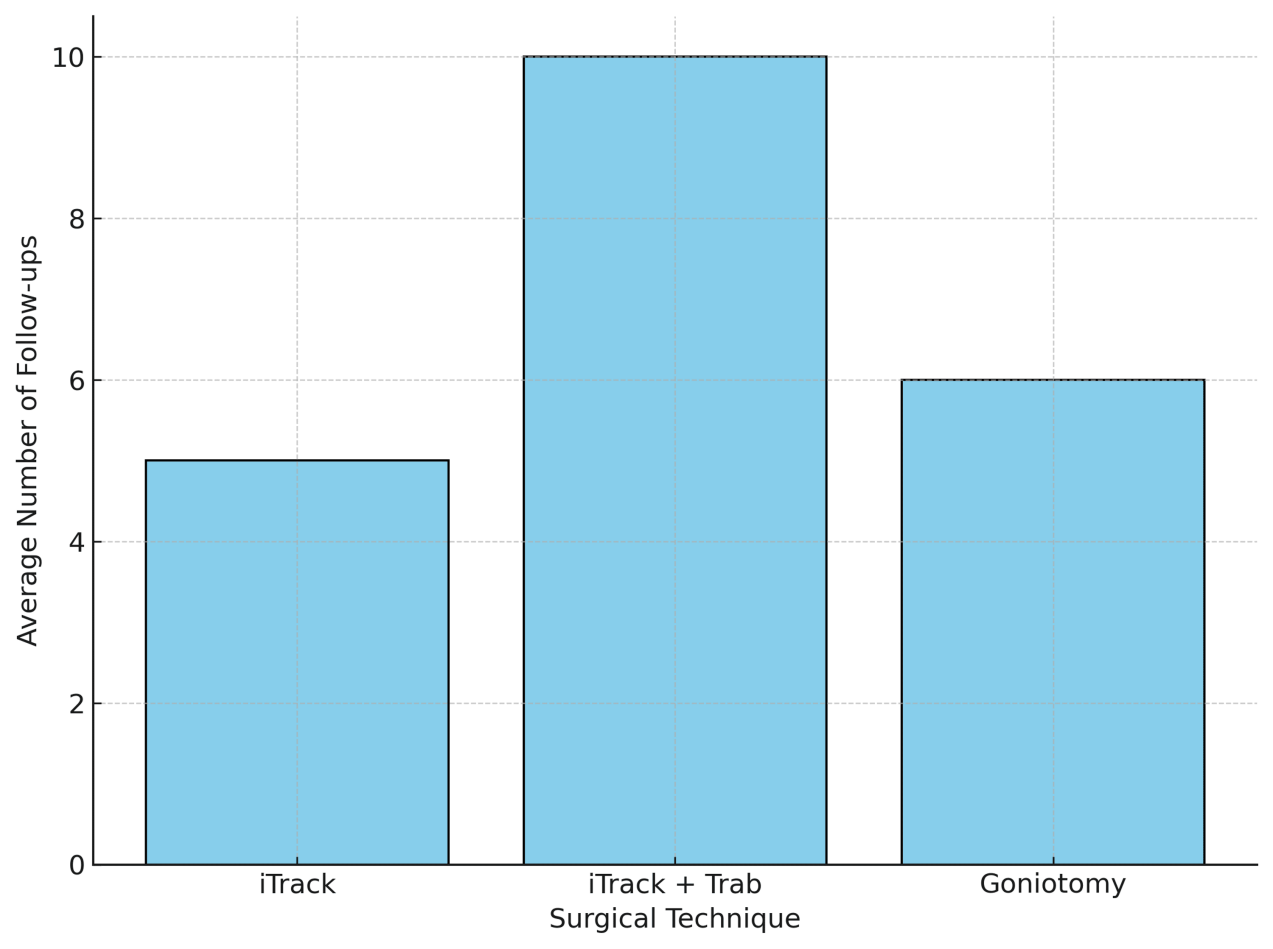
Number of follow-ups per eye, including repeated EUA, in the first two postoperative months EUA: examination of eyes under anaesthesia

Postoperatively, there was a significant reduction in IOP, along with stabilisation or reduction in corneal diameter. Optic disc cupping reversed in 24 out of 31 eyes (77%). The mean IOP reduction was higher for 360° trabeculotomy (62.5%) compared to trabeculectomy (56.6%), with fewer postoperative drops required in the iTrack group. Only two eyes required further glaucoma surgery; these cases likely represented advanced disease at presentation or incomplete initial canalisation. Figure 1 shows the number of follow-ups per eye in the first two postoperative months. The mean follow-up duration was 18 months (range: 6-36 months). No significant intraoperative or early postoperative complications were recorded. On average, the unit received three to four new PCG referrals per year.

## Discussion

Our findings support prior studies indicating that timely surgical intervention in PCG leads to substantial IOP reduction and favourable anatomical outcomes. Male predominance and bilateral involvement in our cohort are consistent with global epidemiology [1,6,8].

The low reoperation rate (6.5%) and reversal of optic cupping in 77% of eyes emphasise the value of early detection and consistent postoperative monitoring [1,4,10,11]. The increased use of iTrack over time reflects broader trends toward microcatheter-assisted 360° trabeculotomy [6,7,9,10].

However, this study has limitations. The retrospective design limits control over variables and excludes functional vision outcomes such as visual acuity, amblyopia, and refractive status. Furthermore, inferential statistical comparisons were not conducted due to sample size limitations. Although the study was conducted at a single UK tertiary centre, the results are relevant to similar high-resource settings; however, generalisability may be limited in lower-resource environments where surgical technology or expertise differs.

## Conclusions

This five-year review from a tertiary centre in the United Kingdom demonstrates that angle-based surgery, particularly 360° trabeculotomy, offers effective IOP control and favourable anatomical outcomes in children with PCG. With timely diagnosis, consistent surgical protocols, and structured follow-up, most patients in our cohort avoided the need for reoperation. However, the absence of functional vision data, such as visual acuity and refractive error, limits the ability to draw conclusions about long-term visual rehabilitation. Future research should aim to include comprehensive functional outcomes and patient-reported quality-of-life measures to support holistic care in paediatric glaucoma management.
